# Risk of Pleural Recurrence in Early-Stage Non-small Cell Lung Cancer in Patients Treated With Surgery vs Stereotactic Ablative Radiotherapy

**DOI:** 10.7759/cureus.73544

**Published:** 2024-11-12

**Authors:** Horiana Grosu, Manuel Cabrera, Daniella Lizarraga Madrigal, Paula Valeria Sainz Zuniga, David Ost

**Affiliations:** 1 Pulmonary Medicine, MD Anderson Cancer Center, Houston, USA; 2 Internal Medicine, Instituto Tecnologico y de Estudios Superiores de Monterrey, Monterrey, MEX; 3 Pulmonary Medicine, Instituto Tecnológico y de Estudios Superiores de Monterrey, Monterrey, MEX

**Keywords:** biopsy, non-small lung cancer, radiation oncoolgy, recurrence, surgey

## Abstract

Background: Pleural recurrence has been reported after thoracic surgery and radiation treatment, and reports show that the surgery as the first treatment may be associated with an increased incidence of pleural recurrence. The main objective of this study was to compare the incidence of ipsilateral pleural recurrence in patients with non-small cell lung cancer (NSCLC) who underwent surgical resection or curative-intent radiotherapy.

Methods: We performed a retrospective cohort study of patients aged 18 or older with stage I NSCLC who underwent surgical resection or curative-intent radiotherapy at our institution. The primary outcome of interest was an incidence of ipsilateral pleural recurrence at the time of first cancer recurrence. Secondary outcomes were the time of any first recurrence type of biopsy approach and the incidence of pleural recurrence.

Results: Our cohort included 512 patients for whom complete data were available. Of these, 50 (9.7%) patients experienced recurrence and 51 died during the five-year follow-up since their first treatment. There was no difference in the incidence of ipsilateral pleural recurrence (P=0.348), the incidence of any recurrence (P=0.069), or time to first recurrence (P=0.088) between the curative intent surgery and radiotherapy groups. Biopsy type was not associated with recurrence.

Conclusion: There was no difference in the incidence of ipsilateral pleural recurrence between the curative intent surgery and radiotherapy groups.

## Introduction

Lung cancer, the most common form of which is non-small cell lung cancer (NSCLC), is the world’s foremost cause of cancer-related death, and surgical resection is the primary curative treatment [1,2]. Screening with low-dose computed tomography (CT) has made possible the detection of early-stage lung cancer (i.e., stage I) and reduced lung cancer mortality [3]. For pulmonary lesions suspected to be malignant, percutaneous needle biopsy and bronchoscopic transbronchial biopsy are commonly used diagnostic procedures. Transthoracic percutaneous needle lung biopsy is particularly useful for lesions in the lung's periphery. More recent advances in navigational and robotic bronchoscopy combined with real-time CT guidance have improved the sensitivity of bronchoscopy for malignancy in peripheral lesions as well [4].

One potential long-term complication of percutaneous needle biopsy is the embedding of tumor cells along the needle tract, and tumor spread through airspaces has been found to have prognostic implications [5-9]. A recent meta-analysis of six studies evaluated data from 2,394 individual patients and concluded that preoperative transthoracic percutaneous biopsy in patients with stage I lung cancer was positively associated with pleural recurrence (SHR 2.58, 95% CI 1.15-5.78) and negatively associated with survival in patients younger than 55 years (SHR 2.53, 95% CI 1.06-6.05) [10]. The recurrence was thought to be related to tumor dissemination through the needle tract.

However, we know that even in early-stage lung cancer, the post-resection recurrence rates remain high, and complete removal of the tumor needs to be ensured both macroscopically and microscopically, which may be challenging. Pleural recurrence has been reported after thoracic surgery [11,12], and factors such as surgical technique and handling of the tumor during surgery itself are assumed to lead to the dissemination of cancer cells in the pleural space [13]. In an individual case, it is difficult to prove that pleural recurrence was caused by the biopsy approach because pleural recurrence can occur even when the diagnostic procedure does not involve the pleura. Several studies indicate that percutaneous needle biopsy increases the risk of pleural recurrence in completely resected early-stage lung cancer. However, it is unclear if the percutaneous needle biopsy or the surgery itself with tumor manipulation increases this risk, or none of these.

The main aim of this study was to compare the incidence of and time to ipsilateral pleural recurrence in patients with stage I NSCLC who underwent resection or curative-intent radiation treatment and time to ipsilateral pleural recurrence. We hypothesized that surgical resection would result in a higher risk of ipsilateral pleural recurrence.

## Materials and methods

We performed a retrospective cohort study of patients 18 years or older with stage I (IA and IB) NSCLC who underwent surgical resection or curative-intent radiation treatment at our institution from January 1, 2016 to January 1, 2018. We restricted enrollment to January 1, 2018 to allow for at least five years of follow-up from the date of first treatment. We excluded all patients who received adjuvant chemotherapy and those patients who were lost to follow-up. The study was approved by the Institutional Review Board of MD Anderson Cancer Center with the number 2023-0002.

Outcomes

The primary outcome of interest was the incidence of ipsilateral pleural recurrence. Ipsilateral pleural recurrence was defined as a malignant pleural nodule or malignant effusion or both on the ipsilateral side of the primary tumor, at the time of first recurrence. Secondary outcomes were time to any first recurrence and the relationship between the biopsy approach and pleural recurrence incidence. Characteristics associated with the risk of recurrence were evaluated.

Statistical considerations

For demographic and clinical characteristics, we used mean and standard deviation to describe continuous variables distributed normally. We used frequencies for categorical data. For time to recurrence, we used the Kaplan-Meier product limit estimator. For the outcome of time to first recurrence, we censored those patients who died. Univariate cause-specific Cox proportional hazards regression was performed to control for confounders. All variables with a p-value less than 0.2 on univariate analysis were candidate variables for multivariable cause-specific Cox proportional hazards regression.

We accepted a two-tailed p-value < 0.05 as statistically significant for all analyses. STATA (Version 14.0; StataCorp., TX, USA) was used to analyze this study.

## Results

We analyzed a data set that included 512 patients for whom complete data were available. Of these, 50 patients (9.7%) experienced recurrence and 51 patients (9.9%) died during the five years since their first treatment. As expected, older patients who had a smoking history and had a diagnosis of chronic obstructive pulmonary disease and cardiac disease were treated more often with radiation. There was no difference in the incidence of any recurrence in the radiation group (34 (11.7%)) vs surgery group (16 (7.2%) (p=0.069)) or ipsilateral pleural recurrence in radiation group (five (1.72%)) vs surgery group (two (0.90%) (p=0.348)) (Table 1).

**Table 1:** Patient characteristics by first treatment received COPD, chronic obstructive pulmonary disease; NSCLC, non-small cell lung cancer

Total, N = 512	Radiation, N = 290	Surgery, N = 222	P-value
Age, mean + SD	72.87 + 8.39	66.68 + 8.80	P < 0.001
Gender (n and %)			
Male	136 (46.9)	85 (38.2)	P = 0.051
Smoking history (n and %)			
Yes	262 (90.3)	160 (72.1)	P < 0.001
Cardiac disease present (n and %)			
Yes	199 (68.6)	117 (52.7)	P < 0.001
COPD present (n and %)			
Yes	168 (57.9)	93 (41.9)	P < 0.001
Cancer histology			
NSCLC undifferentiated	28 (9.6)	36 (16.2)	
Adenocarcinoma	198 (68.2)	146 (65.7)	
Squamous cell carcinoma	64 (22.1)	40 (18.1)	P = 0.065
Clinical stage			
IA	118 (40.6%)	66 (29.7%)	
IA1	38 (13.1%)	14 (6.3%)	
IA2	58 (20%)	56 (25.2%)	
IA3	31 (7.2%)	39 (17.5%)	
IB	45 (15.5%)	47 (21.2%)	P < 0.001
Type of biopsy			
Percutaneous	234 (80.6%)	182 (81.9%)	
Bronchoscopic	56 (19.3%)	40 (18.1%)	P = 0.710
Any recurrence at 5 years			
Yes	34 (11.7%)	16 (7.2%)	P = 0.069
Recurrence in form of distant metastasis			
Yes	4 (1.4%)	4 (1.8%)	P = 0.732
Recurrence in ipsilateral lung			
Yes	17 (5.8%)	6 (2.7%)	P = 0.130
Recurrence in mediastinal/hilar nodes			
Yes	8 (2.7%)	4 (1.8%)	P = 0.566
Ipsilateral pleural recurrence			
Yes	5 (1.72%)	2 (0.90%)	P = 0.348

There were seven ipsilateral pleural recurrences, two in patients who underwent bronchoscopic biopsy and five in patients who underwent percutaneous approach type biopsy, with no significant difference between these groups (P=0.348) (Table 1). No differences in ipsilateral pleural recurrence were seen between biopsy types in patients who received surgery as their first treatment (P=0.237) or radiation as their first treatment (P=0.270). In the surgery group patients, one patient had a bronchoscopic approach and one percutaneous, while in the radiation group, all patients had a percutaneous approach, though this did not reach statistical significance.

On univariate Cox analysis, female gender (HR 0.566, 95% CI 0.323-0.991, P=0.047) and surgery as first treatment (HR 0.507, 95% CI 0.279-0.920, P=0.026) were associated with the lower risk of recurrence. Squamous histology (HR 3.392, 95% CI 1.152-9.998, P=0.027) was associated with an increased risk of recurrence.

All variables with P≤0.20 on univariable analysis were included in multivariable analysis. These variables were female gender, presence of cardiac disease, smoking, cardiac disease, COPD, cancer histology, stage, and first treatment. On multivariate Cox analysis, we found that none of the variables reached statistical significance for risk of recurrence (Table 2).

**Table 2:** Time to any first recurrence COPD, chronic obstructive pulmonary disease; NSCLC, non-small cell lung cancer

Covariate		Univariate model					Multivariate model				
	HR	95% CI			P-value		HR	95% CI			P-value
Age	0.999	0.968		1.031	0.977						
Female	0.566	0.323		0.991	0.047		0.651	0.367		1.154	0.142
Smoking	2.187	0.866		5.552	0.098		1.090	0.393		3.022	0.868
Cardiac disease present	1.858	0.963		3.582	0.064		1.437	0.726		2.844	0.297
COPD present	1.739	0.968		3.123	0.064		1.388	0.723		2.66	0.323
Cancer histology											
NSCLC undifferentiated	1.000										
Adenocarcinoma	1.400	0.489		4.00	0.530		1.236	0.427		3.575	0.695
Squamous cell carcinoma	3.392	1.152		9.981	0.027		2.481	0.808		7.617	0.112
Clinical stage											
IA	1.000						1.00				
IA1	1.174	0.437		3.156	0.750		1.334	0.485		3.668	0.576
IA2	0.938	0.421		2.089	0.877		1.039	0.464		2.327	0.924
IA3	0.366	0.850		1.579	0.178		0.438	0.100		1.909	0.272
IB	1.516	0.756		3.080	0.250		1.477	0.704		3.099	0.301
Type of biopsy											
Bronchoscopic	1.000										
Percutaneous	1.151	0.539		2.456	0.716						
First treatment											
Radiation	1.00						1.00				
Surgery	0.507	0.279		0.920	0.026		0.614	0.327		1.154	0.130

## Discussion

We conducted a retrospective study to assess whether surgery as the first treatment increases the incidence of ipsilateral pleural recurrence and the time to recurrence. We found no difference in ipsilateral pleural recurrence between the surgical and radiation groups. We also found, that compared to bronchoscopic biopsy, transthoracic needle biopsy was not associated with a higher risk of ipsilateral pleura recurrence, regardless of first treatment.

Our findings of low recurrence are similar to prior studies showing that patients with stage I NSCLC treated with surgery had recurrence rates ranging from 6% to 10% per person-year during the first four years; locoregional recurrence was seen in 5% to 10%, and distant metastasis occurred in 10% to 20% [14,15]. This risk of recurrence is similar to that of stereotactic body radiation therapy, [16], though in our cohort we found that surgery as the first treatment modality carried a decreased risk of recurrence in univariate analysis, yet this was not found to be statistically significant in multivariate analysis.

Our findings regarding pleural recurrence were unlike those reported by Hong et al., whose systematic review and individual patient-level meta-analysis found the mode of biopsy to be associated with increased pleural recurrence [10]. In addition, in a study that evaluated 335 patients with stage I NSCLC, of whom 220 were diagnosed by bronchoscopy and 66 by percutaneous needle biopsy, 10 patients experienced pleural recurrence [5]. Of these 10 patients, seven had undergone needle biopsy performed with an 18-gauge cutting needle. The proportion of patients developing pleural recurrence was greater for patients diagnosed via percutaneous needle biopsy than for those diagnosed via bronchoscopy (9.1% versus 1.0%, P<0.0028) [5]. Similarly, Inoue et al. explored the clinical outcomes of 447 patients with stage I lung cancer [7]. A CT-guided percutaneous core needle biopsy was performed in 131 (29.3%), transbronchial biopsy in 175 (39.1%), and open lung biopsy in 141 (31.5%) patients. Local recurrence with pleural dissemination occurred in eight of the 131 (6.1%) patients who underwent percutaneous biopsy and in only five of the 316 (1.6%) who underwent transbronchial biopsy or open lung biopsy (P<0.01) [7]. However, recurrence did not affect disease-free survival [7]. Similar findings were reported in two other retrospective studies, which concluded that the diagnostic transthoracic needle biopsy procedure was associated with pleural recurrence but was not associated with recurrence-free survival in patients with early-stage lung cancer [8,9]. The differences between our study and the above studies may be explained by the low number of patients in our cohort, and significant limitations of the prior studies related to the retrospective approach, studies from Asian countries, with crucial information such as follow-up time and staging data missing.

We recognize that needle tract tumor dissemination is probable with any needle biopsy and in an experimental study evaluating the pleural dissemination in lung lobes resected during treatment of lung cancer in patients who underwent fine needle aspiration of the tumor demonstrated positive malignant cells in 60% of pleura-irrigated specimens after fine needle biopsy, compared to 10% positive malignancy before fine needle biopsy: after resection. However, the clinical implication of this was unclear and a recent study that evaluated tumor spread through airspace, concluded that prognosis was not influenced by the preoperative biopsy [6,17].

Our study also has limitations such as the retrospective nature of data collection, which carries a potential for bias. In addition, our study population received care at a dedicated cancer center, which probably represents a self-selection of patients with a greater desire to seek interventions compared with the general population. Another important limitation is the sample size, especially as the risk of recurrence is relatively low in this patient population, and our study could be underpowered to detect a small difference. A better approach to answer this complex question would be to clarify the real risk of needle biopsy concerning the tumor seeding and needle track implantation, mode of surgery, and risk of tumor seeding during surgical resection, and the clinical implications this would have on the risk of recurrence.

## Conclusions

In conclusion, surgery as the first treatment did not show an increased association with the risk of ipsilateral pleural recurrence in stage I NSCLC. A better approach to answer this complex question would be to clarify the real risk of needle biopsy concerning the tumor seeding and needle track implantation, mode of surgery, and risk of tumor seeding during surgical resection, and the clinical implications of this on the risk of recurrence. In addition, a larger study with more patients could detect a difference.
